# Genome sequence and characterization of a novel *Pseudomonas putida* phage, MiCath

**DOI:** 10.1038/s41598-023-48634-z

**Published:** 2023-12-09

**Authors:** James Jaryenneh, Joseph S. Schoeniger, Catherine M. Mageeney

**Affiliations:** Sandia National Laboratories, Livermore, CA 94550 USA

**Keywords:** Microbiology, Bacteriophages, Genome

## Abstract

*Pseudomonads* are ubiquitous bacteria with importance in medicine, soil, agriculture, and biomanufacturing. We report a novel *Pseudomonas putida* phage, MiCath, which is the first known phage infecting *P. putida* S12, a strain increasingly used as a synthetic biology chassis. MiCath was isolated from garden soil under a tomato plant using *P. putida* S12 as a host and was also found to infect four other *P. putida* strains. MiCath has a ~ 61 kbp double-stranded DNA genome which encodes 97 predicted open reading frames (ORFs); functions could only be predicted for 48 ORFs using comparative genomics. Functions include structural phage proteins, other common phage proteins (e.g., terminase), a queuosine gene cassette, a cas4 exonuclease, and an endosialidase. Restriction digestion analysis suggests the queuosine gene cassette encodes a pathway capable of modification of guanine residues. When compared to other phage genomes, MiCath shares at most 74% nucleotide identity over 2% of the genome with any sequenced phage. Overall, MiCath is a novel phage with no close relatives, encoding many unique gene products.

## Introduction

*Pseudomonas putida* is a gram-negative bacteria that can be found in most soils and water and includes many members that can colonize plant roots and provide benefits to the root microbiomes^1^. *Pseudomonas putida* strains have been widely explored for use in biomanufacturing platforms, bioremediation, biocontrol, and as a factory for natural products^2^. Few bacteriophages have been isolated that infect *P. putida*^3–5^. Phages targeting *P. putida* are of interest not only for understanding viral ecology of *Pseudomonads*, but also potential biotechnology applications, such as manipulating *P. putida* populations in the rhizosphere^6^, degrading biofilms through phage-encoded depolymerase enzymes^7^ or potentially as vectors for delivery of large genetic cassettes into *P. putida* genomes.

Here we report the first phage isolated against *P. putida* S12, MiCath. MiCath has a myovirus morphology, is a virulent phage, and has a ~ 61 kbp dsDNA genome encoding a unique queuosine biosynthesis pathway, endosialidase, and a cas4 exonuclease. MiCath has a broad host range infecting five diverse strains with average nucleotide identity (ANI) ranging from 90.2 to 99%. Further, MiCath designates a new genus and species of phage taxonomy since the nearest phage relative only shares 2% of its genome with MiCath. Overall, MiCath represents a novel phage that could have application spaces in biomanufacturing, bioremediation, and biocontrol.

## Results and Discussion

### MiCath isolation, morphology, and characterization

**Figure 1:**
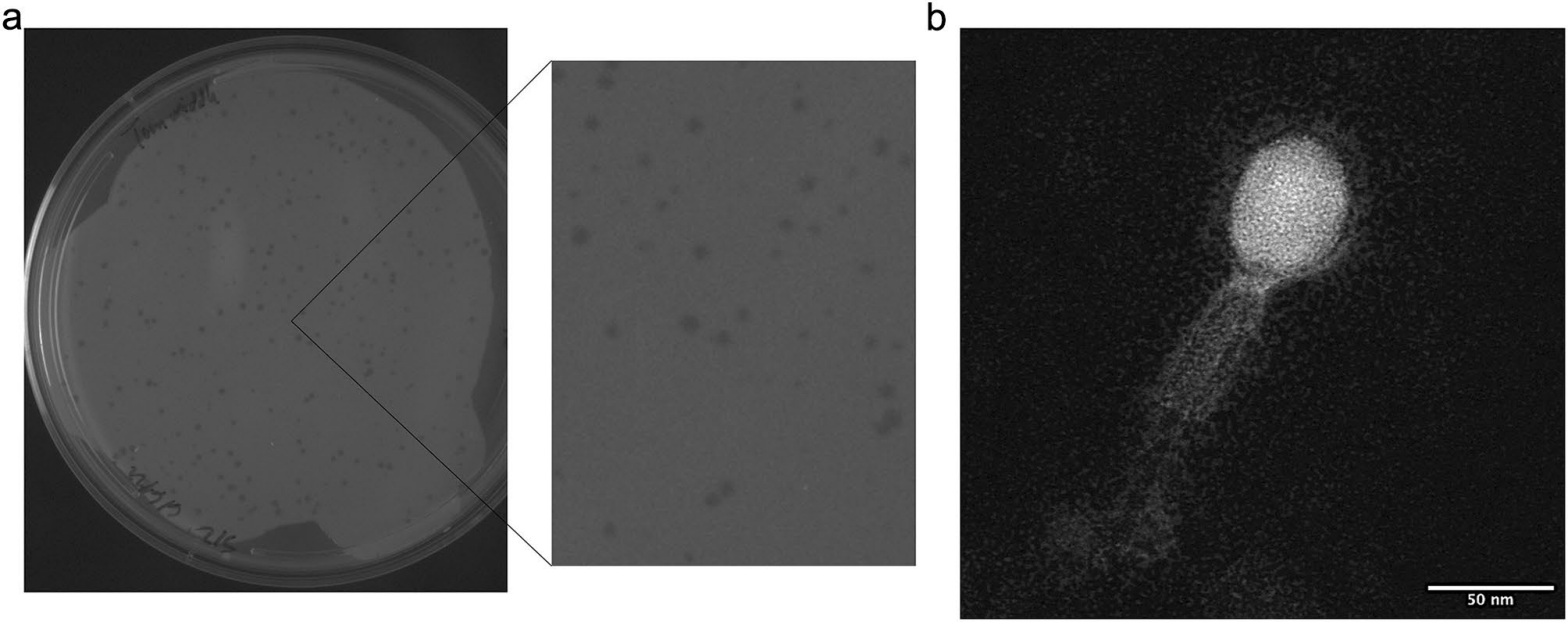
MiCath isolation and morphology. (**a**) MiCath 1 mm clear plaques on *P. putida* S12. (**b**) TEM image of MiCath revealing an A1 myovirus morphotype. Grids were stained with 10% gadolinium acetate tetrahydrate.

Bacteriophage MiCath was isolated from soil obtained from a home garden under a tomato plant using the enrichment plating method. MiCath produced clear, 1mm plaques on *P. putida* S12 after 16 h at 30 °C (Fig. 1a). A high titer lysate of MiCath was produced, grids were prepared and stained with 10% gadolinium acetate tetrahydrate and used for transmission electron microscopy (TEM) imaging. MiCath has an A1 myovirus morphology^8^ (Fig. 1b). The capsid measures: height 58.1 nm (± 1.5 nm), width 45.4 nm (± 2.8 nm), and the tail measures 123.5 nm (± 2.1 nm) (n = 5).

### MiCath infection kinetics

**Figure 2:**
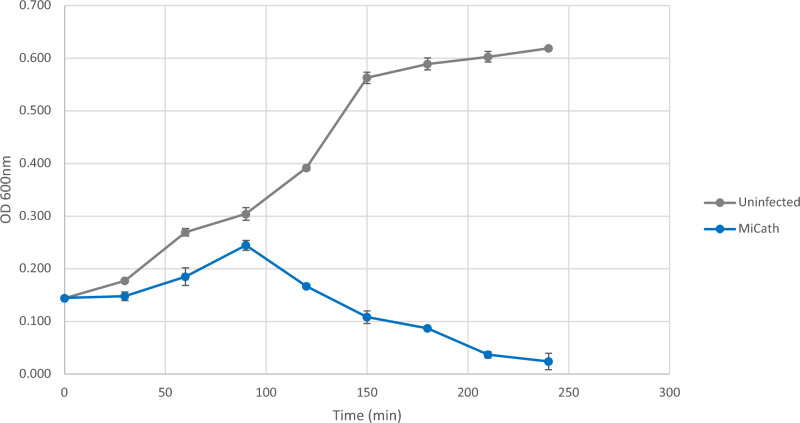
Lysis timing for MiCath. Lysis assays were completed in triplicate for MiCath using *P. putida* S12 as the host. MiCath begin lysis around 30 min with nearly complete lysis observed by 240 min. Error bars represent one standard deviation. N = 3.

We sought to explore infection kinetics typically tested for bacteriophages for MiCath. We explored burst size and lysis infection timing. The burst size for MiCath is 18 ± 5 (n = 3); this burst size is small compared to ~ 60 which is the phage average^9^. However, many of the phages included in the average are coliphages, which have different growth conditions than many environmental bacteria such as *P. putida* and different growth kinetics than phages of other bacterial genera^10^. MiCath begins lysis around 30 min, overt lysis is observed after 90 min, and at 240 min significant lysis of the culture is observed (Fig. 2). Despite its smaller burst size, it is competent and can quickly kill a culture within a few hours.

### MiCath host range

To determine the host range of MiCath, seven additional *P. putida* strains and *P. aeruginosa* PAO1 were obtained, tenfold dilutions of MiCath were spotted onto a lawn of each host tested, and efficiency of plating (EOP) was calculated. MiCath infects 5 strains in two different GTDB^11^ species, *Pseudomonas_E hunanensis* and *P. putida*, suggesting MiCath can infect multiple strains of *P. putida*, including those traditionally used in biomanufacturing applications (Table 1). *Pseudomonas putida* EM383 is a prophage-null mutant^12^ of *P. putida* KT2440. Notably, MiCath can infect both *P. putida* KT2440 and *P. putida* EM383, yet this occurs at different EOPs. There are at least two possible reasons MiCath infects the prophage-laden KT2440 strain more efficiently. There could be unknown defense mechanisms in EM383 that are either repressed by the prophages found in KT2440 or broken by prophage integration. Alternatively, prophage encoded proteins in KT2440 may enhance the lytic capabilities of MiCath. Further work would be needed to reveal more detail of interactions between the prophages in KT2440 and the incoming MiCath DNA.

**Table 1:** Host range of MiCath on *P. putida* strains.

Host bacteria	GTDB species	Genome assembly number	Infectivity	Efficiency of plating (EOP)
*Pseudomonas putida* S12	*hunanensis*	GCA_000495455.2	Yes	1
*Pseudomonas putida* KT2440	*hunanensis*	GCA_000007565.2	Yes	10^–1^
*Pseudomonas putida* EM383	*hunanensis*	GCA_000007565.2	Yes	10^–3^
*Pseudomonas putida* F1	*hunanensis*	GCA_000016865.1	Yes	10^–1^
*Pseudomonas putida* DOT-T1E	*hunanensis*	GCA_000281215.1	No	0
*Pseudomonas putida* NCTC10936	*putida*	GCA_900455645.1	Yes	10^–3^
*Pseudomonas putida* JUb85	*putida_Q*	GCA_004345845.1	No	0
*Pseudomonas putida* p106	*putida_E*	GCA_003936645.1	No	0
*Pseudomonas aeruginosa PAO1*	*aeruginosa*	GCA_000006765.1	No	0

Strains tested are listed in order of phylogenetic distance from *P. putida S12.* Strain name, GTDB species, NCBI assembly number, infection outcome, and EOP are noted.

### MiCath genome characterization

**Figure 3:**
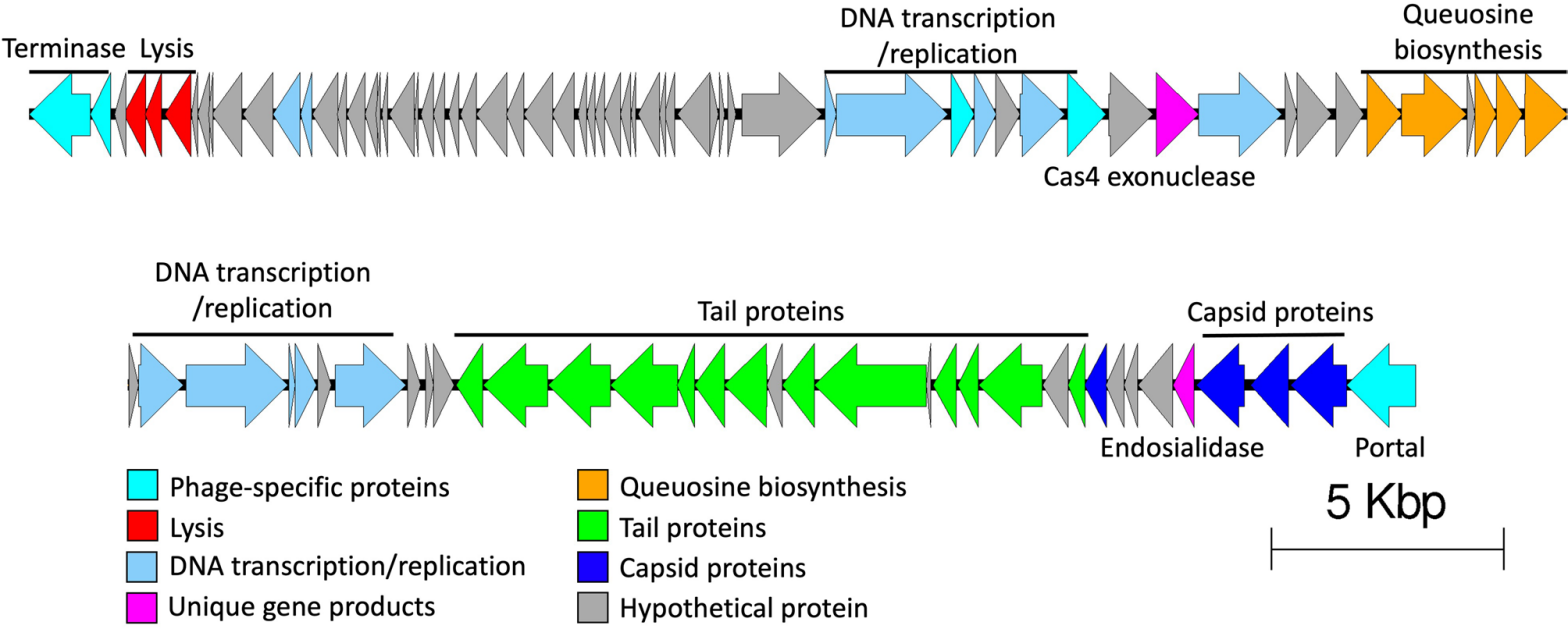
Genomic characterization of MiCath. MiCath has a 60,958 bp dsDNA genome that encodes 97 ORFs. The families of functional protein calls are colored coded by function as denoted in the figure.

Sequencing and de novo assembly revealed MiCath has a double-stranded DNA genome of 60,958 bp and has 97 predicted open reading frames (ORFs) (Fig. 3). The function of 48 ORFs was predicted by comparing the protein sequences against previously annotated phages using BLASTP^13,14^ and against known hidden Markov Models (HMMs) using the HHpred server^15,16^. There were 49 ORFs (50.5%) for which function could not be predicted using these annotation tools.

MiCath does not encode an integrase gene and forms clear plaques suggesting it is a virulent phage not capable of integration. We observed ORFs with high percent amino acid identity to other traditional phage genes that encode tail, capsid, terminase, and lysis proteins. MiCath also encodes an entire cassette dedicated to queuosine biosynthesis, suggesting it is capable (in certain contexts dependent on expression mechanisms) to alter its DNA nucleotides, as seen in other phages, protecting its genome from host restriction systems^17^. MiCath also encodes an endosialidase, which may be responsible for the broad host range observed^18^. Lastly, MiCath has a predicted ORF with a high percent amino acid identity to cas4 exonucleases. This is not uncommon in phage genomes: previous work reports cas endo/exonucleases in many genomes^19^; however, the exact function of cas endonucleases in phage genomes are still unknown. All proteins and putative functions are listed in Supplemental Table 1.

### MiCath phylogenetic analysis

MiCath has nearly no nucleotide sequence similarity to any previously sequenced phage. The closest relative is a *Xanthomonas* phage vB_Xar_IVIA-DoCa3 (Genbank accession number: ON911540) with 74.9% identity over 2% of the genome. There are three regions of identity found with other phages including parts of the queuosine biosynthesis pathway, the major capsid protein, and a hypothetical protein. Overall, MiCath only shares any nucleotide identity with 13 additional phages infecting bacteria in the genera: *Pseudomonas*, *Pantoea*, *Escherichia*, and *Sphingomonas* and three partial phage metagenome assemblies genomes from humans (Genbank Accession Numbers: BK020071.1, BK039317.1, and BK047545.1).

**Figure 4:**
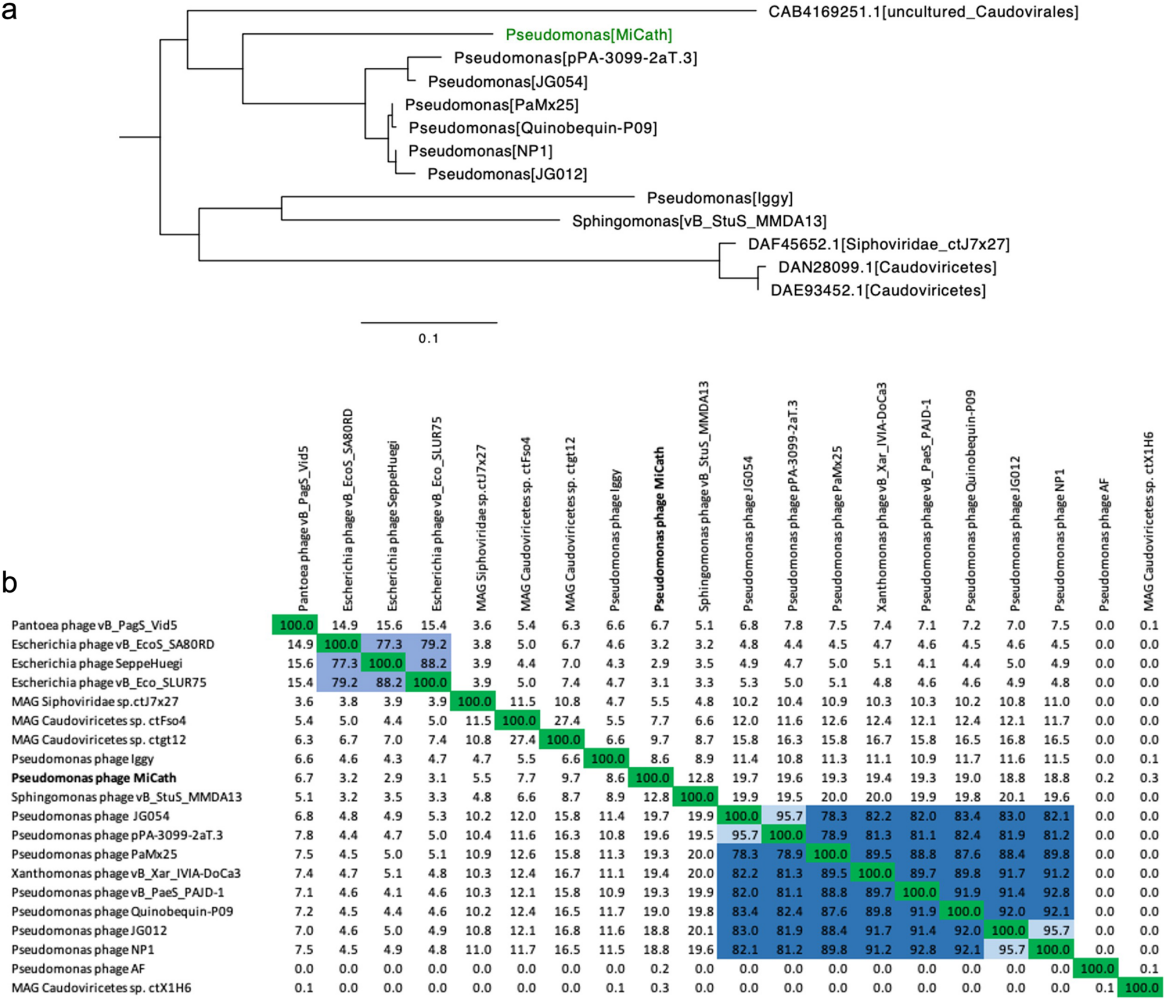
Phylogenetic analysis of MiCath. (**a**) Protein phylogenetic tree of clade containing MiCath and other phages major capsid proteins. FastTreeDBL^22^ was used to calculate the tree using the Le-Gascuel 2008 model^44^. Image is a portion of the full tree depicted in Supplementary Fig. 1. (**b**) Heatmap of pairwise intergenomic relatedness adapted from VIRIDIC^23^ output between phage genomes found in the same major capsid protein clade (Fig. 4a). Green indicates the same genome with 100% relatedness, genus clusters are in blues (70% relatedness) and species clusters are in light blue (95% relatedness). MiCath is bolded.

Since the full nucleotide sequence was not similar to any phages, we performed phylogenetic analysis on the major capsid protein (GenBank: WAX22437.1), which is identified in most phage genomes and has been used previously for phylogenetic analysis^20,21^. There were 62 additional phage proteins with high percent amino acid identity to the major capsid protein encoded by MiCath. We aligned these homologous proteins and created a phylogenetic tree using FastTreeDBL^22^. This analysis revealed that MiCath encodes a similar major capsid protein to other *Pseudomonas* phages but is the most distant member of the *Pseudomonad* clade (Fig. 4a; Supplemental Fig. 1). Pairwise average nucleotide analysis with the phages encoding a major capsid protein in the same clade as MiCath was computed using VIRIDIC^23^ (Fig. 4b) and shows MiCath shares little nucleotide relatedness with these phages, similar to the result from the entire phage landscape. The criteria from the International Committee on Taxonomy of Viruses states the genera cut off is 70% nucleotide identity and species is 95% nucleotide identity, suggesting MiCath denotes a new phage genus and species within the class *Caudoviricetes* and subfamily *Queuovirinae*^24,25^.

### MiCath DNA resists restriction enzyme digestion

Many phages have been discovered whose genomes encode queosine biosynthesis pathway genes^17,26–28^. Recently, this gene cluster was shown in *Enterobacteria* phage 9G to modify up to 27% of the guanine nucleotides in its genome to a hyper modfied guanine, 2′-deoxyarcheosine (dG+)^29^. Additionally, it was shown that 9G and additional phages containing the altered dG+ nucleotides were resistant to restriction enzymes, suggesting an anti-defense mechanisms encoded by this gene cluster^17^.

**Figure 5:**
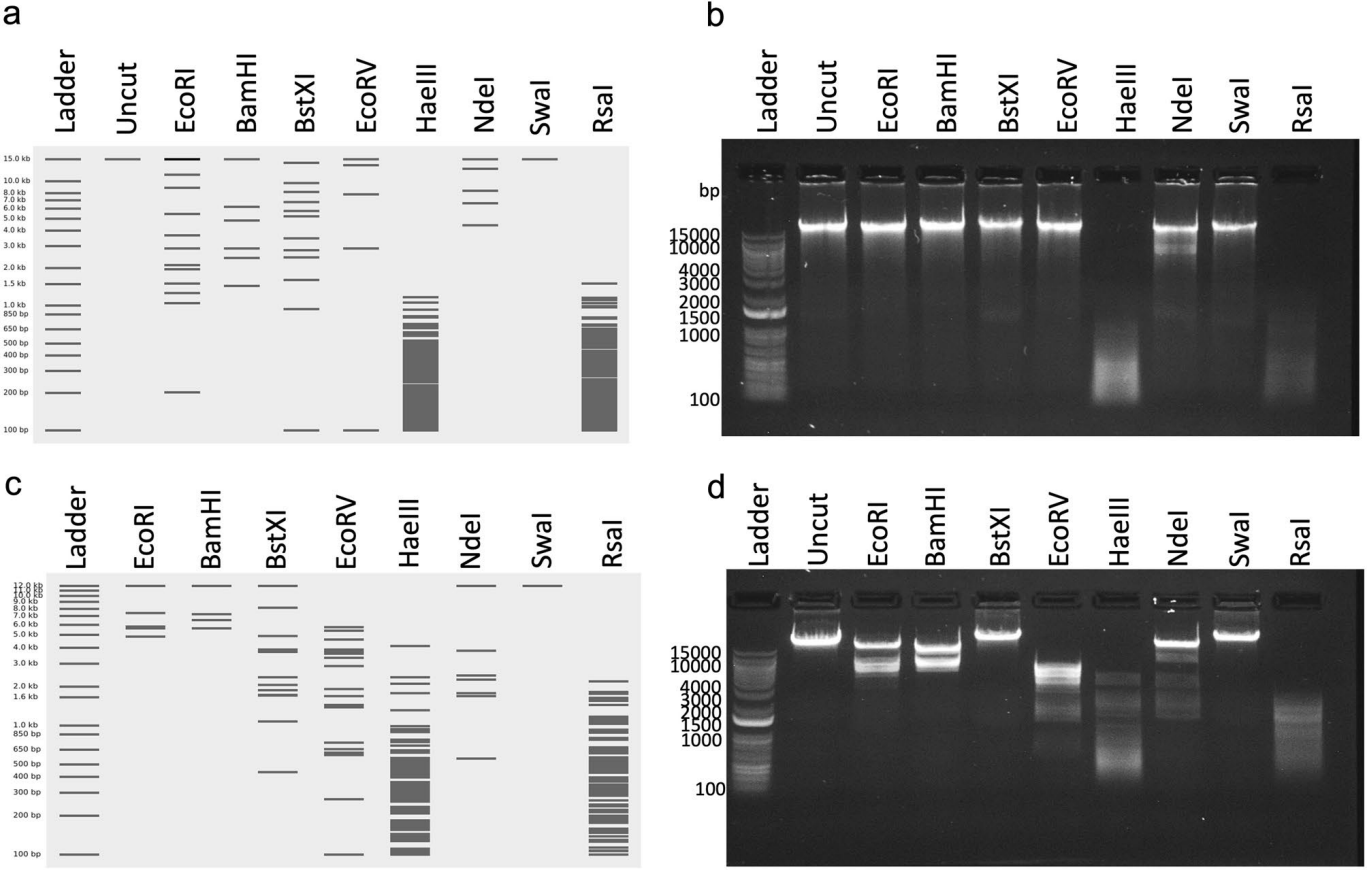
Restriction patterns for MiCath DNA. (**a**) MiCath DNA virtual restriction digests for enzymes used, prepared by Benchling online software. (**b**) 0.8% agarose gel after MiCath DNA was digested for 60-min with EcoRI, BamHI, BstXI, EcoRV, HaeIII, NdeI, SwaI, RsaI restriction enzymes. (**c**) Lambda DNA virtual restriction digest for enzymes used, prepared by Benchling online software. (**d**) 0.8% agarose gel after commercially available Lambda phage DNA was digested for 60-min with EcoRI, BamHI, BstXI, EcoRV, HaeIII, NdeI, SwaI, RsaI restriction enzymes. The ladder used on both agarose gels in (**b**) and (**d**) is Invitrogen 1 Kb DNA Plus, the highest molecular weight band on the ladder is 15 kb.

We sought to detemine through restriction enzyme analysis if the queuosine biosynethesis gene cluster present in MiCath might be capable of incoporating dG+ or a similar modified based into DNA. We digested MiCath DNA with a panel of restriction enzymes previously used to explore queuosine biosynthesis pathways in phages^17^. We used an online tool, benchling^30^, to virtually digest the MiCath genome with each enzyme to confirm expected results, indicating seven of the eight selected enzymes should digest MiCath’s unmodified genome (Fig. 5a). We performed restriction digestions and found that SwaI doesn’t cut MiCath’s genome as predicted. However, EcoRI, BamHI, and EcoRV also do not cut MiCath’s genome suggesting protection of the MiCath genome from restriction by these enzymes (Fig. 5b). Restriction to EcoRI is consistent with the presence of dG+ or a related modification, as previously reported^17,29^. Further, partial digestion was observed for BstXI, while complete digestion is observed for HaeIII, NdeI, and RsaI (Fig. 5b). The resistance to these restriction enzymes, coupled with the complete functional gene set to produce dG+ (DpdA, folE, QueD, QueE, and QueC [MiCath genome coordinates 28869-33191]) observed in other phages^17^ suggest MiCath is likely modifings its genome with dG+ nucleotides to prevent restriction digestion by its host. As a control we also digested commercially available Lambda DNA (Fig. 5d) with the same panel of restrition enzymes and the pattern matches that generate by virtual digestion (Fig. 5c).

## Conclusions

Here we report the discovery of the first phage isolated and sequenced for *P. putida* S12, MiCath. To date only 23 phages have been discovered for any *P. putida* strains^3,4^ indicating a gap in phage biology of this important species, and a potential need for phages suitable for use in various applications. MiCath is a virulent phage with a novel genome not similar to any previously sequenced phages. MiCath also encodes many novel genes within its genome that may allow for broad host range, alternative DNA base usage, and insights into phage-encoded cas endonucleases. Overall, MiCath represent an interesting new phage that may also be useful for bioremediation and biomanufacturing applications.

## Methods

### Bacterial strains

We used *P. putida* strains S12, DOT-T1E, F1 (kindly gifted by Grant Rybnicky), ATCC 12633 (purchased from ATCC), JUb85 (kindly provided by Samuel Buck), EM383 (kindly gifted by Huseyin Tas), p106 (kindly provided by Carey-Ann Burnham), and KT2440 (obtained from lab stocks). An overnight culture of each *P. putida* was prepared in Luria–Bertani (LB) broth and incubated at 30 °C overnight shaking at 250 rpm for all experiments.

### Environmental sample collection and phage filtrate preparation

Soil was collected from a backyard garden (37.93132569198107, -121.7123907950711) under a tomato plant. 3–5 mL of soil and 10 mL of SM phage buffer (SMPB) (100 mM NaCl, 8 mM MgSO_4_·7H_2_O, 25 mM Tris-HCl [pH 7.4]) were mixed vigorously by inverting. The 15 mL tube was then allowed to settle for at least 20 min followed by filtration through a 0.22 µm filter.

### Enrichment plating

The enrichment method was used to isolate phages from the soil sample. The soil filtrate was mixed 1:1 with LB broth and 20 uL of overnight *P. putida* S12, incubated overnight, shaking at 250 rpm at 30 °C. The overnight culture was centrifuged at 845×*g* for 3 min and the supernatant was filtered through a 0.22 µm filter. Enrichment filtrates were tested for evidence of phage using a traditional double agar overlay^31^. Briefly, 100 uL of the enrichment filtrate and 100 uL of *P. putida* S12 were incubated 10 min then 3 mL molten top agar was added, plated onto LB agar plates, and incubated at 30 °C overnight.

### Phage purification and lysate creation

To isolate a single phage, an individual plaque was picked with a sterile pipette tip into SM phage buffer and plated using double agar overlays and incubated at 30 °C overnight. This process was repeated three times to ensure a pure phage population.

A lysate was created by flooding a webbed plate (near complete lysis) with 8 mL of SM phage buffer, incubating for 30 min at room temp and filtering through a 0.22 µm syringe filter.

### Host range testing

To determine the host range of MiCath, the phage lysate was titered using a tenfold serial dilution in SMPB and spot titered on several *P. putida* bacterial strains. Bacterial lawns for each tested bacteria were prepared from overnight cultures using the double agar overlap technique. 3 uL of each phage dilution was spotted onto the bacterial lawn and allowed to sit for 15 min at room temperature followed by overnight incubation at 30 °C. After incubation, plaques were counted on the lowest countable dilution, the phage titer was calculated followed by calculation of efficiency of plating.

$${\text{Titer }}\left( {{\text{pfu}}/{\text{mL}}} \right) = \left( \# {\text{ pfu}}/{\text{volume used in}} \,\upmu{\text{l}} \right) \times \left( 10^{3} \upmu{\text{l}}/{\text{ml}} \right) \times {\text{ dilution}}\;{\text{ factor}}$$

$${\text{EOP}} = {\text{titer on test strain}}/{\text{titer on}}\;P.{\text{ }}putida\;{\text{S}}12$$

### Transmission electron microscopy

Phages were imaged by transmission electron microscopy (TEM) on a Themis Z transmission electron microscope operated in scanning transmission electron microscopy (STEM) mode. Fresh phage lysates > 10^9^ pfu/mL were applied to a carbon grid (Ted Pella, catalog no. 1813) and incubated at room temperature for 10 min and wicked off with Whatman filter paper. The grids were washed twice with 10uL of diH_2_O for 2 min wicking off in between. The grid was then stained with 10 uL of uranyl acetate alternative stain (Ted Pella, catalog no. 19485, 10% gadolinium acetate tetrahydrate) for 10 min and wicked dry. Grids were allowed to completely dry in a chemical fume hood for 1 h and stored until imaging.

TEM images were visualized and analyzed in ImageJ software^32^ using the TIA plugin. Five images were selected for measurement analysis. Three independent measurements of the width and height of each capsid and the tail from capsid to end of tail spike were measured. The scale bar was also measured independently three times, averaged, and corrected to 50 nm (if needed). Each capsid or tail average was corrected based on the scale bar correction. The capsid and tail measurements were averaged, and standard deviation was calculated for all five images overall.

### Burst size analysis

Burst size analysis was completed as previously described^33^. Briefly, 500 uL of an overnight *P. putida* S12 was inoculated into 9.5 mL of LB broth and incubated shaking at 30 °C until the OD_600_ reached 0.6. 1 mL of MiCath (corresponding to 10^6^ phages, MOI = 10) was added and incubated at 30 °C without shaking for 5 min (Culture 1). After 5 min 100 uL of culture 1 was transferred to a new flask with 10 mL of fresh LB broth (Culture 2). Culture 2 was incubated for 30 seconds and 1 mL was transferred to a microcentrifuge tube for analysis. Immediately 20 uL was titered to determine the non-adsorbed titer (titer 1). Then chloroform was added to the original microcentrifuge tube and supernatant was removed and titered (titer 2). Culture 2 was incubated at 30 °C for 12 min and 1 mL was removed and chloroform treated, and supernatant was removed to obtain the free phage titer (titer 3). Phage titers at each step were performed by serial dilution of the phage lysate and double agar overlays on *P. putida* S12. The titer was calculated as previously described, followed by burst size calculation shown below.

$${\text{Initially Infected Cells }}\left( {{\text{N}}_{{\text{i}}} } \right) = {\text{Titer}}1{-}{\text{Titer}}2$$

$${\text{Burst Size}} = {\text{Titer}}3 - {\text{N}}_{{\text{i}}}$$

### Lysis assay

Overnight *P. putida* S12 was diluted to OD_600_ = 0.1 and split into six culture flasks. Three cultures were infected with MiCath at MOI = 10, the remaining three were left uninfected. The flasks were incubated shaking at 30 °C for 4 h, measuring the OD_600_ every 30 min.

### Genome sequencing and analysis

Genomic DNA was isolated from the phage lysates through DNA extraction using Promega wizard Kit (Promega, catalog no. 0000457646) following the manufacturer protocol. A library was prepared using the Nextera DNA prep Kit (Illumina, catalog no. FC-121-1031) following the manufacturers recommended protocol and sequenced on a NextSeq 500/550 using 150-bp cycles high output kit (Illumina, catalog no. 20024907). The sequencing run produced 3,855,168 reads with a Q30 of 96.50%.

Sequencing reads were processed with BBDuk^34^ to remove low quality reads using the following parameters: ktrim = r, k = 21, mink = 11, hdist = 1. After filtering there were 3,854,915 total reads with a Q30 of 96.51%. FastQC^35^ was used to evaluate the quality of reads before and after filtering with standard parameters. Filtered reads were assembled with SPAdes (v3.9.0)^36^. Any scaffold with less than 10% of the top scaffold coverage and smaller than 1000bp was removed from the assembly. The average coverage was 8667 × across the entire genome. Bowtie2^37^ was used to align 95.74% (3,690,663 reads) of the raw filtered reads back to the final MiCath genome.

Coding sequences (CDS) were predicted by GeneMarkS^38^ and command-line multiPhATE v0.5 ^39^ with Glimmer v3.02^40^, Prodigal v2.6.3^41^, and Phanotate v0.13.0^42^ gene callers turned on. The open reading frames (ORFs) predicted were functionally annotated by searching amino acid sequences against BLASTP^13,14^ standard databases and HHpred (using PDB_mmCIF70 and Pfam-A_v35 databases)^15,16^.

### Phylogenetic analysis

Phylogenetic analysis was completed using proteins with high percent amino acid identity to the major capsid protein. The protein sequences that had any blastP high percent amino acid identity with the predicted major capsid protein of MiCath were collected from NCBI. The sequences were aligned with MUSCLE v3.8.31^43^ using standard options. A phylogenetic tree was made with FastTreeDBL v2.1.10^22^ using the Le-Gascuel 2008 model^44^ using standard options. Final trees were visualized using FigTree v1.4.4 (http://tree.bio.ed.ac.uk/software/figtree/).

We obtained the following complete and partial genome sequences from NCBI to compute intergenomic relatedness for the phages with major capsid proteins in the same clade as MiCath: *Pseudomonas* phage AF (NC_019923.1), *Pseudomonas* phage Iggy (NC_070970.1), *Pseudomonas* phage JG012 (KX898399.1), *Pseudomonas* phage JG054 (NC_072498.1), *Pseudomonas* phage NP1 (KX129925.1), *Pseudomonas* phage PaMx25 (NC_041953.1), *Pseudomonas* phage pPA-3099-2aT.3 (OP784576.1), *Pseudomonas* phage Quinobequin-P09 (NC_072501.1), *Pseudomonas* phage vB_PaeS_PAJD-1 (NC_072500.1), *Escherichia* phage vB_Eco_SLUR75 (LR025197.1), *Escherichia* phage vB_EcoS_SA80RD (OL960575.1), *Escherichia* phage SeppeHuegi (MZ501104.1), *Pantoea* phage vB_PagS_Vid5 (NC_042120.1), *Sphingomonas* phage vB_StuS_MMDA13 (NC_072503.1), *Xanthomonas* phage vB_Xar_IVIA-DoCa3 (NC_072499.1), Caudoviricetes sp. isolate ctFso4 (BK039317.1), Caudoviricetes sp. isolate ctgt12 (BK047545.1), Siphoviridae sp. ctJ7 × 27 (BK032517.1), Caudoviricetes sp. isolate ctX1H6 (BK020071.1). We used VIRIDIC^23^ web version with standard options to calculate whole genome nucleotide identity using a single multiFASTA of these phage genome sequences.

### Restriction digestion

To isolate high quality MiCath DNA, a MiCath phage lysates was incubated overnight at 4 °C with a 2:1 lysate to PEG (1M NaCl, 20% PEG8000) ratio. The overnight PEG precipitation was centrifuged at 10,000×*g* for 30 min at 4 °C and the supernatant was discarded. The resulting pellet was resuspended in 1 mL of dH_2_0. DNA was extracted using the Norgen Biotek Corp Kit (Norgen, catalog no. 46800) following manufacturer’s protocol including DNase I step followed by DNase inactivation and eluted in diH_2_0.

MiCath and Lambda (NEB, catalog no. N3011S) DNAs were digested with EcoRI-HF (NEB, catalog no. R3101S), BamHI-HF (NEB, catalog no. R3136S), BstXI (NEB, catalog no. R0113S), EcoRV-HF (NEB, catalog no. R3195S), HaeIII (NEB, catalog no. R0108T), NdeI (NEB, catalog no. R0111S), SwaI (NEB, catalog no. R0604S), RsaI (NEB, catalog no. R0167S). Restriction enzymes digestions were prepared by combining 10 × NEB reaction buffer, 500 ng of MiCath DNA, sterile H_2_O, and 1 uL of restriction enzyme. The tube was briefly flicked to mixed and centrifuged before incubating at 37 °C for 1 h. The reaction was briefly spun and loaded and run on a 0.8% agarose gel.

Virtual DNA digests and virtual gels were created using Benchling^39^. The FASTA sequence for MiCath was input into the software and enzymes were selected and a virtual gel was created.

### Supplementary Information

Supplementary Information.

## Data Availability

MiCath whole genome sequence has been deposited at NCI Genbank under the accession number OP882271.
