# Supplementary material for: Genome sequence and characterization of a novel Pseudomonas putida phage, MiCath
Source: Sci Rep. 2023 Dec 9;13:21834. doi: 10.1038/s41598-023-48634-z (PMC10710427; doi:10.1038/s41598-023-48634-z)
Supplement: Supplementary file 1 — Supplementary Information. [file 41598_2023_48634_MOESM1_ESM.pdf]

Genome sequence and characterization of a novel *Pseudomonas putida* phage, MiCath

James Jaryenneh[1], Joseph S. Schoeniger[1], Catherine M. Mageeney[1*]

Supplemental Information

**Left coordinate**	**Right Coordinate**	**Strand**	**Predicted Function**
1	1326	-	terminase, large subunit
1323	1763	-	terminase, small subunit
1847	2086	-	hypothetical protein
2043	2522	-	Lysin B
2506	2856	-	holin
2936	3493	-	Lysin A
3502	3645	-	hypothetical protein
3645	3899	-	hypothetical protein
3869	3973	-	hypothetical protein
3942	4589	-	hypothetical protein
4611	5264	-	hypothetical protein
5261	5851	-	mazG-like nucleotide pyrophosphohydrolase
5862	6101	-	DNA-directed RNA polymerase
6117	6662	-	hypothetical protein
6646	6849	-	hypothetical protein
6852	7247	-	hypothetical protein
7244	7513	-	hypothetical protein
7482	7571	-	hypothetical protein
7610	7741	-	hypothetical protein
7774	8307	-	hypothetical protein
8315	8395	-	hypothetical protein
8453	8725	-	hypothetical protein

8718	8966	-	hypothetical protein
9047	9277	-	hypothetical protein
9304	9648	-	hypothetical protein
9645	10310	-	hypothetical protein
10291	10671	-	hypothetical protein
10668	11291	-	hypothetical protein
11288	11758	-	hypothetical protein
11841	12029	-	hypothetical protein
12038	12211	-	hypothetical protein
12208	12423	-	hypothetical protein
12399	12758	-	hypothetical protein
12755	12979	-	hypothetical protein
12989	13093	-	hypothetical protein
13134	13598	-	hypothetical protein
13600	13734	-	hypothetical protein
13715	13933	-	hypothetical protein
13992	14681	-	hypothetical protein
14680	14865	+	hypothetical protein
14895	15005	+	hypothetical protein
15077	15241	+	hypothetical protein
15381	17093	+	hypothetical protein
17171	17419	+	DNA-binding protein
17416	19812	+	primase/helicase
19885	20388	+	Host-nuclease inhibitor protein Gam
20388	20861	+	RNaseH
20854	21375	+	hypothetical protein
21372	22337	+	DNA ligase
22397	23230	+	AAA ATPase

23296	24249	+	hypothetical protein
24317	25225	+	cas4 family CRISPR-associated exonuclease
25225	27024	+	DNA helicase
27091	27357	+	hypothetical protein
27357	28091	+	hypothetical protein
28189	28791	+	hypothetical protein
28869	29603	+	putative queuosine biosynthesis protein QueE
29609	31012	+	putative queuosine biosynthesis protein QueC
31016	31195	+	hypothetical protein
31188	31652	+	putative queuosine biosynthesis protein QueD
31652	32212	+	GTP cyclohydrolase I
32271	33191	+	Queuine tRNA-ribosyltransferase
33191	33397	+	hypothetical protein
33397	34365	+	DNA polymerase III
34423	36621	+	DNA polymerase B
36639	36773	+	Exonuclease
36776	37240	+	Holliday junction resolvase
37261	37551	+	hypothetical protein
37637	39169	+	DNA Helicase
39205	39477	+	hypothetical protein
39591	39767	+	hypothetical protein
39764	40204	+	hypothetical protein
40245	40823	-	tail fiber assembly protein
40831	42228	-	tail collar protein
42240	43580	-	minor tail protein
43577	45034	-	baseplate protein
45012	45395	-	baseplate wedge protein
45392	46036	-	putative spike protein

46033	46956	-	baseplate hub protein
46953	47288	-	hypothetical protein
47291	47980	-	minor tail protein
47977	50394	-	tape measure protein
50394	50495	-	hypothetical protein
50537	51037	-	tail assembly chaperone protein
51078	51515	-	Tail tube protein
51524	52891	-	major tail protein
52905	53459	-	hypothetical protein
53456	53815	-	head-to-tail adaptor protein
53812	54288	-	phage morphogenesis protein
54275	54658	-	hypothetical protein
54660	54950	-	hypothetical protein
54960	55706	-	hypothetical protein
55718	56167	-	endosialidase
56226	57260	-	Major capsid protein
57387	58208	-	minor structural protein
58262	59461	-	minor capsid protein
59461	60948	-	portal protein

Supplemental Table 1 Genome Characterization of MiCath. Location of predicted open reading frames, strand encoded on + is forward strand, - is reverse strand, and predicted function.

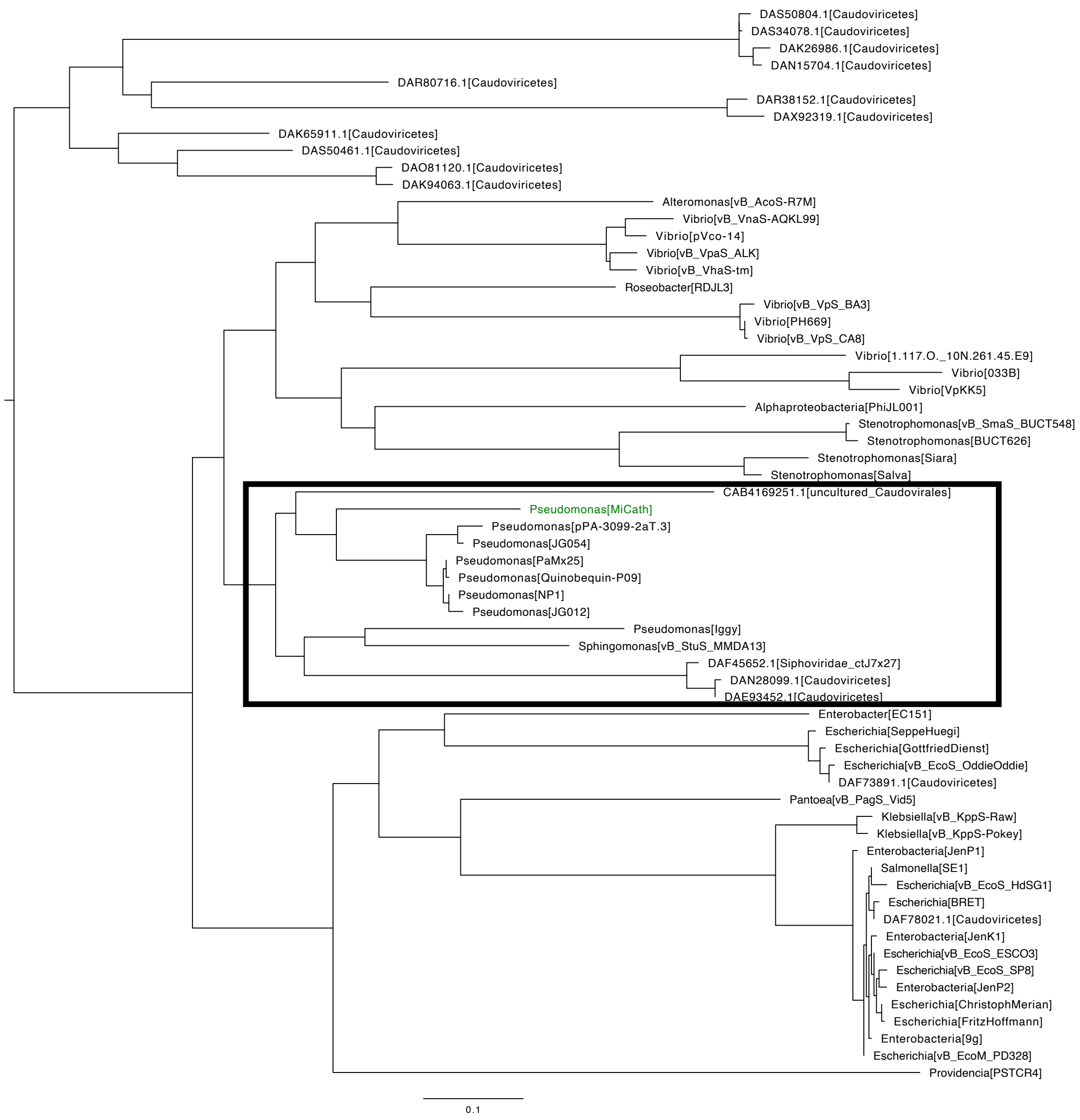

Supplemental Figure 1. Major capsid protein tree of MiCath and all homologous major capsid proteins. Figure 4A major capsid tree for MiCath and *Pseudomonas* clade were extracted from this image (black box). All major capsid proteins were aligned with MUSCLE[40], tree were made using FastTreeDBL[22], and viewed in FigTree.
